# Occipital Lobe Cavernoma Presenting With Headaches and Visual Hallucinations: A Case Report

**DOI:** 10.7759/cureus.51506

**Published:** 2024-01-02

**Authors:** Mohammed A Abdulaal, Fatema M Ali, Zainab H Rabea, Alaa M Husain, Zainab A Naser, Sajeda K Mohamed, Zainab A Abdulla, Ahlam Alharbi

**Affiliations:** 1 Emergency Medicine, Dallah Hospital, Riyadh, SAU; 2 General Practice, Al Hilal Multi Specialty Medical Center, Hamad Town, BHR; 3 General Practice, RAK (Ras Al Khaimah) Medical & Health Sciences University, Ras Al Khaimah, ARE; 4 General Practice, Taj Medical Center, Budaiya, BHR; 5 Medicine, Wenzhou Medical University, Wenzhou, CHN; 6 Dentistry, Wenzhou Medical University, Wenzhou, CHN; 7 Medicine, Mansoura University, Mansoura, EGY; 8 Family Medicine, Primary Health Care Center, Riyadh, SAU

**Keywords:** computed tomography, magnetic resonance imaging, occipital lobe, headache, visual hallucination, cavernoma

## Abstract

Cavernomas, also known as cavernous angiomas or cavernous malformations, are rare vascular lesions characterized by abnormal clusters of dilated capillaries without intervening brain tissue. While often asymptomatic, they can manifest with neurological symptoms such as headaches, seizures, and focal deficits. We present a case of a 45-year-old male who presented with persistent headaches and visual hallucinations. Thorough clinical assessment revealed intermittent throbbing headaches localized to the left occipital region, accompanied by brief episodes of vivid visual hallucinations. Extensive work-up, including laboratory tests and neuroimaging, identified a subependymal cavernoma in the left occipital lobe. A surgical excision was performed, resulting in sustained relief from headaches and the absence of visual hallucinations during follow-up examinations. This case contributes to the understanding of cavernomas by detailing the clinical presentation, diagnostic process, and successful surgical intervention for a subependymal cavernoma in the left occipital lobe. The resolution of symptoms postoperatively underscores the importance of individualized treatment approaches, considering factors such as lesion location, patient age, and associated risks. The case highlights the evolving knowledge in cavernoma management and emphasizes the need for tailored therapeutic decisions in addressing neurovascular disorders.

## Introduction

Cavernomas, also known as cavernous angiomas or cavernous malformations, are vascular lesions characterized by clusters of abnormally dilated capillaries without intervening brain tissue. They represent a relatively uncommon but clinically significant neurovascular abnormality. The majority of cavernomas are asymptomatic; however, when symptomatic, they can manifest with a range of neurological symptoms, including headaches, seizures, and focal neurological deficits [1].

In the context of headaches and visual hallucinations, the case presented here highlights the intricate clinical presentation of a patient ultimately diagnosed with a subependymal cavernoma located in the left occipital lobe. This case underscores the importance of a systematic and multidisciplinary approach in the evaluation and management of patients with such neurological conditions. The integration of detailed clinical history, comprehensive physical examination, and neuroimaging techniques played a pivotal role in achieving an accurate diagnosis and guiding subsequent therapeutic interventions [1,2].

## Case presentation

A 45-year-old male patient presented to the neurology clinic with a chief complaint of persistent headaches associated with visual hallucinations over the past six months. The patient reported experiencing intermittent throbbing headaches primarily localized to the left occipital region, accompanied by episodes of visual hallucinations characterized by vibrant, colorful shapes and patterns. The visual hallucinations were described as brief, lasting for a few minutes, and were not associated with any specific triggers. The patient reported no other associated symptoms such as nausea, vomiting, or photophobia during the headache episodes.

The medical history revealed no significant past medical illnesses or neurologic disorders. The patient denied any recent trauma or head injuries. Family history was unremarkable for neurological conditions. Additionally, there was no history of substance abuse, including alcohol and recreational drugs. The patient's social history revealed a non-smoker.

On physical examination, the patient appeared well-nourished and in no apparent distress. Vital signs were within normal limits. Neurological examination revealed no focal motor or sensory deficits. Cranial nerve examination was normal, and fundoscopic examination revealed no papilledema, hemorrhages, or exudates. The remainder of the physical examination, including cardiovascular, respiratory, and abdominal systems, was unremarkable.

Given the nature of the symptoms, a comprehensive work-up was initiated. Laboratory investigations, including complete blood count with differential, electrolyte panel, renal and liver function tests, thyroid function tests, and inflammatory markers (erythrocyte sedimentation rate and C-reactive protein), were within normal limits. Serological tests for infectious etiologies such as syphilis and human immunodeficiency virus were negative (Table 1).

**Table 1:** Laboratory investigations of the patient

Lab test	Result	Reference range
Hemoglobin (Hb)	14.2 g/dL	12.0-16.0 g/dL
White blood cell (WBC) count	8,200/mm³	4,000-11,000/mm³
Platelet count	280,000/mm³	150,000-450,000/mm³
Sodium (Na)	141 mmol/L	135-145 mmol/L
Potassium (K)	4.2 mmol/L	3.5-5.0 mmol/L
Renal function tests	Within normal limits	
Blood urea nitrogen (BUN)	16 mg/dL	8-20 mg/dL
Serum creatinine	0.9 mg/dL	0.6-1.2 mg/dL
Free thyroxine (T4)	1.1 ng/dL	0.8-1.8 ng/dL
Erythrocyte sedimentation rate (ESR)	12 mm/hour	0-20 mm/hour
C-reactive protein (CRP)	1.2 mg/L	0-3.0 mg/L
Syphilis (rapid plasma reagin)	Non-reactive	
Human immunodeficiency virus (HIV)	Non-reactive	

Furthermore, a lumbar puncture was performed to analyze cerebrospinal fluid. The cerebrospinal fluid analysis revealed normal opening pressure, clear appearance, and normal cell count. Protein and glucose levels were within the normal range, with negative results for infectious markers, including bacterial cultures.

**Figure 1:**
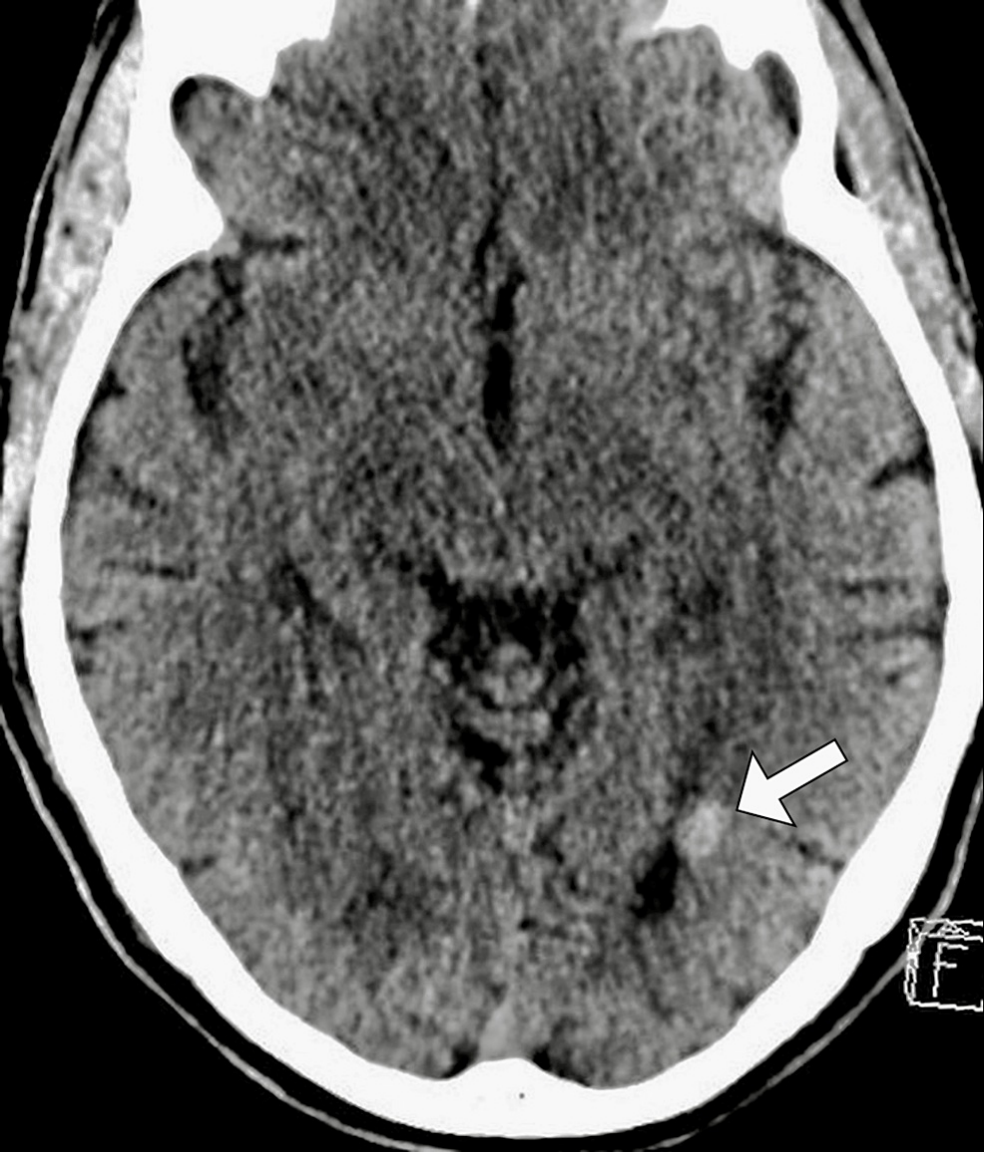
Axial CT of the head revealing a hyperdense nodule (arrow) adjacent to the occipital horn of the left lateral ventricle.

**Figure 2:**
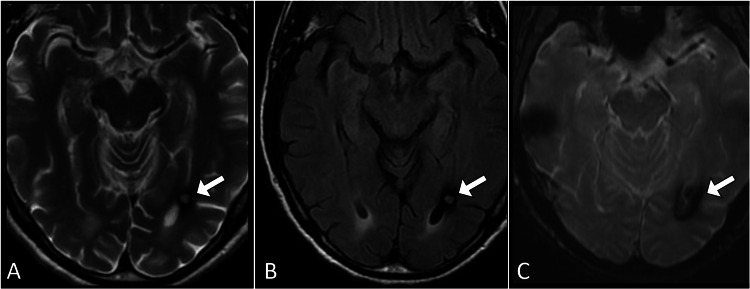
Axial MRI brain images including T2-weighted (A), FLAIR (B), and gradient echo (C) revealing a subependymal lesion (arrow) at the occipital horn of the left lateral ventricle. The lesion exhibits high signal intensity on T2 and FLAIR (A and B) with a surrounding rim of low signal intensity. Additionally, the gradient echo image (C) displays a blooming artifact, characteristic of cavernoma. FLAIR: fluid-attenuated inversion recovery.

Imaging studies were conducted to further investigate the cause of the patient's symptoms. A computed tomography scan of the brain revealed a hyperdense focus in the subependymal location of the occipital horn of the left lateral ventricle (Figure 1). Subsequent magnetic resonance imaging of the brain with contrast confirmed the presence of a subependymal cavernoma. The lesion appeared hyperintense on FLAIR (fluid-attenuated inversion recovery) imaging with a characteristic rim of hypointensity, consistent with cavernoma features. The cavernoma measured approximately 1.2 cm in diameter and was close to the occipital horn of the left lateral ventricle (Figure 2).

The patient underwent surgical intervention for the subependymal cavernoma. The neurosurgical procedure involved a craniotomy for excision of the lesion. Intraoperatively, the cavernoma was carefully dissected from surrounding neural tissue and excised. The patient tolerated the procedure well without any immediate complications. The patient has been closely monitored in the neurology clinic for postoperative outcomes. The patient has reported sustained relief from headaches, and the visual hallucinations have not recurred. Neurological examinations during follow-up visits have remained unremarkable.

## Discussion

Cavernomas, also known as cavernous angiomas or cavernous malformations, represent vascular lesions characterized by clusters of abnormally dilated capillaries devoid of intervening brain tissue. While a majority of cavernomas are asymptomatic, symptomatic cases can present with a spectrum of neurological symptoms, including headaches, seizures, and focal neurological deficits [1,2]. In our presented case, the clinical manifestation of headaches and visual hallucinations prompted an extensive diagnostic work-up, incorporating laboratory investigations and advanced neuroimaging, ultimately confirming the presence of a cavernoma.

Magnetic resonance imaging serves as a cornerstone in evaluating cerebral cavernous malformations, providing distinctive findings across various sequences. Cerebral cavernous malformations typically exhibit hypointensity on T1-weighted images, while T2-weighted images reveal characteristic hyperintensity attributed to hemosiderin deposits from chronic hemorrhages. FLAIR sequences enhance lesion visibility, particularly near ventricular spaces. Susceptibility-weighted imaging plays a pivotal role, showcasing hypointense foci indicating hemosiderin presence, thereby offering high sensitivity for lesion detection. Contrast-enhanced magnetic resonance imaging contributes to understanding hemodynamics and vascular relationships [1-3]. Serial magnetic resonance imaging examinations remain crucial for monitoring changes in lesion size, detecting new hemorrhages, and guiding treatment decisions over time [4].

While the precise pathogenesis of cavernomas remains unclear, a notable proportion is associated with genetic mutations. Identified mutations in three genes - CCM1 (KRIT1), CCM2 (MGC4607), and CCM3 (PDCD10) - contribute to our understanding of the genetic basis of cavernomas, facilitating risk assessment and holding promise for future targeted therapies [2-4].

The management of cavernomas is contingent on various factors, including patient age, symptoms, lesion location, and associated risks. Conservative management, surgical resection, and radiosurgery are among the available treatment options, with decisions carefully considering the risks and benefits associated with each approach [3-5]. Surgical excision, exemplified in our case, remains a crucial option for symptomatic cases, demonstrating successful outcomes and contributing valuable insights to the ongoing discourse on optimal management strategies. The resolution of symptoms postoperatively in our case aligns with existing literature advocating for surgical intervention in select cases. This emphasizes the importance of individualized therapeutic decisions and contributes to the evolving understanding of cavernoma management.

## Conclusions

In conclusion, the presented case of a subependymal cavernoma in the left occipital lobe underscores the complexity of diagnosing and managing neurovascular disorders. Through a clinical assessment, advanced neuroimaging, and targeted surgical intervention, this case contributes to the expanding knowledge of the varied clinical presentations of cavernomas, specifically highlighting the association with headaches and visual hallucinations. The successful resolution of symptoms postoperatively, coupled with the consideration of conservative management, highlights the importance of individualized approaches in treating cavernomas.
